# Hyperpyrexia: Its Causes, Prognosis, and Treatment

**Published:** 1907-03-23

**Authors:** 


### SPECIAL ARTICLE.

### HYPERPYREXIA: ITS CAUSES, PROGNOSIS, AND TREATMENT.

### 6. The Methods of Treating Hyperpyrexia.

The method of treating hyperpyrexia is, *par excellence*, the extraction of heat from the surface of the patient by the external application of cold. It is true that there are many drugs which will reduce pyrexia—notably phenacetin, 10 to 15 grain doses; phenazone, 10 to 20 grain doses; acetanilide, 4 to 8 grain doses; tincture of aconite; aspirin, 10 grain doses; and quinine;—but the effects of the drugs have been found in most cases to be more serious than are those of the hyperpyrexia itself. These drugs exert a very dangerous action upon the heart; it is even possible that a considerable part of their antipyrexial action depends upon their depression of the circulation, for it is a well-known clinical fact that heart-failure tends towards low temperature. There are a few conditions, however, in which drugs may safely be used to check the fever from rising to a great height; amongst these are, first, malaria, in which quinine or quinine sulphate should be given in large doses by the mouth in ordinary cases, but intramuscularly into the buttocks if the condition is urgent; for intramuscular injections the best form of quinie is the lactate, which is soluble in water to the extent of 1 in 10; from 15 minims to ʒj. of the 10-per cent. solution may be injected at a time, all the usual aseptic precautions being strictly observed; secondly, influenza, in which it is generally held to be dangerous to resort to cold sponging and the like during the acute attack, and in which either quinine in the form of drachm doses of the ammoniated tincture, two hourly, or phenazone in two doses of 10 or 15 grains each at an interval of eight hours, or aspirin in 10 grain doses, four hourly, are most likely to do good; and thirdly, lobar-pneumonia, in which the fever is often reduced if the patient can be got to sleep; and in which, therefore, a 10 or even 15 grain dose of pulv. ipecac. co. (Dover's powder) will often do all that is required.

Upon the whole, however, the treatment of hyperpyrexia, and of pyrexia which may be high and yet not reach 106° F., is by the external application of cold. It remains to describe in detail the various methods we have of doing this. Some of the following are obviously, in private practice, easier than others; some are not possible except in exceptional circumstances, and with wealthy patients; but all are given because there are different occasions upon which each and every one of them may be of service:—

1. *Tepid Sponging*: This is one of the easiest procedures, in that in most cases it requires but one nurse or attendant, and necessitates the use of nothing which is not likely to be at hand. Two blankets and a flannel nightgown opening down the back are put before the fire to warm, and two or three hot bottles are got ready for use presently. A large mackintosh sheet is warmed; a convenient basin is filled with water at a temperature of about 90° F.; a large sponge and two warm rough towels complete the preparations. The patient being gently rolled as far over to one side of the bed as may be, the mackintosh sheet is spread over the draw-sheet, if possible covering the entire bed. The patient is then gently rolled back on to the mackintosh, and the nightgown, and all the bedclothes over him except a blanket, are removed. This blanket, of course, gets wet, but it is removed later on. The nurse then wrings the sponge out of the water, making it but moderately dry, and then gently but firmly sponges the head, face, and neck first, and afterwards passing the sponge beneath the blanket, the front of the trunk, the arms, and the legs. The sponge is rewrung out of the water now and then, and each time the temperature of the water goes down a little, reaching perhaps 70° F., or even 65° F., according to the temperature of the room. Having sponged the front of the body and limbs, the nurse gently rolls the patient half over on the mackintosh and sponges the back of the head, trunk, and limbs, from above downwards in a similar way, until the whole body, back and front, has been carefully sponged. Care should be taken that there is no draught across the bed at the time—that is to say, the bed should not be directly between the fireplace and an open window, the fireplace and a door ajar, or an open window and a door ajar. The whole sponging takes about ten minutes or a quarter of an hour. The patient is then rolled over to the far side of the bed again, the wet mackintosh is removed; with a warm absorbent towel the patient is rapidly rubbed over, it being unnecessary to dry the skin thoroughly; one of the fresh hot blankets is laid upon the bed under him, he is rolled gently back on to it, the damp blanket over him is replaced by another hot dry one, the warm flannel nightgown is put on; and lest the extremities have become unduly cold so as to prevent sleep, a hot bottle wrapped in flannel is placed to each foot, with another possibly by the side of the legs. All this takes but a few minutes; the nurse then brings the second warm towel and completes the drying of the hair (in a woman it is unnecessary to wet the long hair); takes the temperature, which will very likely have fallen two to three degrees; perhaps gives the patient some warm fluid to drink, and leaves him to sleep. One of the best influences of a sponging of this kind is the sleep it brings in its train; this sleep should on no account be disturbed. Should the temperature have again reached a dangerous height, when the patient awakes the sponging may be repeated in exactly the same way; it may sometimes be necessary to sponge the patient as often as four or five times in the 24 hours.

2. *Cold Sponging*: This is done in exactly the same way as tepid sponging, the only difference being that instead of starting with water at 90° F. and allowing it to cool, cold water (65° F. to 40° F.) is used from the beginning. In the summer-time, and in robust males, this is sometimes preferred; but the sudden application of cold to the body is unsuitable for quite young or for elderly patients, particularly if they be females.

3. *Block Ice*: Instead of sponging, the somewhat heroic treatment of passing a slab of block ice all over the body from above downwards is occasionally employed. Although it sounds heroic, and even cruel, this is not really the case. Strong men with high temperature in lobar-pneumonia, for example, enjoy the application of ice to the body, and it acts more rapidly in reducing the temperature than does sponging. The *modus operandi* is similar to that described for sponging; the disadvantages of the method are, first, that block ice is not obtainable in many instances; and secondly, that the patient's friends may think the doctor cruel, even though the patient really likes the treatment.

4. *Ice Poultices*: These also can only be used when ice is obtainable; they disturb the patient but little, and do not necessitate any changing of the bed-clothes, but they have the disadvantage of being applicable to small portions of the body only, so that the amount of heat abstracted by them is neither great nor rapid, and therefore they do not reduce pyrexia to anything like the extent that tepid or cold sponging or cold baths do. In some cases, however, they are very useful, notably in lobar pneumonia in strong persons. To make the poultice, a suitable flat rubber or mackintosh bag is required, and into this a quantity of powdered ice is put. To powder the ice, a strong hat pin or knitting needle serves better than anything else, the block of ice being quickly splintered up when the sharp point of the pin is repeatedly struck slantwise into its surface. When the poultice is worn by the patient the ice melts, and will wet the bed unless the bag into which it is put is securely fastened up by tying or otherwise. It is best to wrap the bag in a thin layer of flannel, and then apply it to the patient's chest, *e.g.*, over the site of the pneumonia, keeping it on with a many-tailed bandage. The poultice should be renewed in about eight hours, when the ice has all melted and the water has become as hot as the patient's body. Many patients prefer an ice poultice to a linseed poultice, but it is for the patient rather than for the doctor to say whether the poultices should be continued. In any case, as has been said, the reduction of temperature to be expected from the poultice is not great.

5. *The Wet Pack*: In country practice, with only unskilled assistance, the wet pack is particularly useful because it is so simple; it is also fairly efficient. Preferably there should be two beds in the room. A large mackintosh sheet is laid over one bed, covering the undersheet; a thin blanket is laid over the mackintosh; a large sheet is then soaked in cold water, wrung out only moderately dry, and placed over the mackintosh; the patient is taken from the other bed, laid on the wet sheet, wrapped up in it, except as to the face, the nightgown having been removed; a thick, hot, dry blanket is then laid over the top of him. Hot bottles may be needed for the feet. He is left in the wet pack for a variable time; if he falls asleep, he may remain till he wakes; otherwise he may be taken out of the wet sheet in an hour's time and put back into his original bed again between hot blankets. The usual effect of the wet pack is to evoke a refreshing perspiration afterwards, except in lobar pneumonia before the crisis; it is the perspiration which helps in reducing the temperature as much as does the actual coldness of the sheet itself. Indeed, though cold at first, the sheet is soon made steamy hot by the patient's body, so that it is only at the beginning that the pack acts by direct withdrawal of heat from the skin.

6. *Ice-bags or buckets in cradle*: This form of treatment is simple, and avoids disturbance to the patient, but it requires a supply of ice renewable from time to time. It has the advantage of not wetting things much, and of being applicable by an untrained person. The patient lies in bed with a thin blanket over him; over this is placed a large cradle, and over the cradle as many more blankets as may be thought wise. The outside blankets should be well tucked in around the edges of the bed and round the patient's neck and feet. It is wise to have hot bottles at the feet. Inside the cradle are slung four or five rubber ice bags, or a similar number of small watertight buckets. Into each bag or bucket, as the case may be, a quantity of ice is put, and this ice need not be broken up small. In this way a cold space is kept, free from draught, in proximity to the patient without disturbing him. When the ice melts it needs renewal, the cradle and ice-bags being used continuously if required. There is one small point which needs attention, namely, the fact that vapour from the patient will condense upon the ice-bags; and, in the course of time, begin to drip down on to the blanket which covers him. This blanket will therefore require changing occasionally.

7. *Leiter's Coils*: A Leiter's coil is simply a continuous coil of thin lead piping, through which cold water can be run. Such a coil can be applied to any part of the body which needs cooling, for example, to the head, one side of the chest, a joint, and so on; but it has several disadvantages in the treatment of pyrexia. In the first place, the lead is heavy, and therefore the coil is limited as to its possible dimensions; secondly, it can only remove heat from a small area of the body at a time; thirdly, it requires a supply of running water, from a tap or syphon, and a tank or sink near the bed for the water to run away into. Leiter's coils are therefore seldom used for the reduction of hyperpyrexia, though frequently used in the cooling of local inflammations, or in the relief of some sorts of headache.

8. *The Warm Bath allowed to Cool*: A Long, deep bath is required, big enough for immersion of the patient's body. Hot blankets, hot bottles, a hot drink, two hot rough towels, and a hot dry flannel nightgown are got ready, as described under tepid sponging. The bath is brought near the bed, or the bed near the bath, so as to avoid carrying the patient far. At least two, and possibly three, assistants are required if he be an adult. The bath is filled with water at about 90° F., or even warmer, so that the patient will not feel a sudden shock on being immersed in it. The assistants then raise the sheet or blanket on which the patient is lying, and thus gently lift him off the bed with as little disturbance as possible, and carry him to the bath. They then lower him, sheet or blanket and all, into the bath, keeping his head raised out of the water. Either now, or just previously, the patient's head and the front of the chest should be sponged with cool water and kept wet. The temperature of the bath is now lowered by pouring in cold water slowly but steadily, the cold being stirred in to mix it with the warm, until the bath thermometer registers 70° F. or 65° F. Some prefer to reduce the bath temperature by putting ice into it, but the ice really has no particular advantage over plain cold water, and the latter costs nothing. The rate of cooling of the bath is regulated by pouring in the cold water less or more quickly as seems good at the time; the patient remains immersed for from quarter of an hour to 25 minutes. He is then lifted in the sheet out of the bath on to a convenient place, such as a couch covered with a mackintosh, where he is rapidly and but partially dried, the wet wrapping being exchanged for a hot dry blanket; a hot nightgown is put on, and the patient is then lifted in the dry blanket back to bed, where he is covered with another hot blanket, or two; hot bottles are placed to his feet, he is given a hot drink, and will probably go off to sleep.

The warm bath which is cooled in this way when the patient is in it, is a most efficient and safe way of reducing the temperature between 1° and 3° F. It is extensively used in cases of typhoid fever, the baths being begun whenever the patient's temperature exceeds 103.5° F., and being repeated twice or more times a day if necessary. The disadvantages of the method are the number of appliances needed and the number of attendants required.

9. *The Cold Bath*: This is given exactly as is the warm bath which is cooled, the only difference being in the initial temperature of the bath. This may be 70° F., 65° F., or 60° F., according to the temperature of the air of the room and the strength of the patient. Whilst the patient is in the bath, the limbs and thorax should be rubbed continually with a sponge, and water colder than that of the bath should be poured over the head every three or four minutes. The bath lasts ten minutes as a rule, but should be discontinued sooner if cyanosis or weakness of the pulse come on. It is in typhoid fever *par excellence* that the cold bath is advocated, and those who use it give it four to six times a day. The bath is contra-indicated when the pulse is intermittent or irregular, or in elderly or quite young people. The puerperal state, pregnancy, bronchopneumonia, pneumonia, and albuminuria are no contra-indications.

10. *Cold Affusion*: This is applicable in cases where it is desired to give a bath, but no bath of the required size is available. It is a method which can be used in country practice, though except in unusual cases it is less likely to be adopted than is tepid sponging or one of the other simpler methods described above. For cold affusion a very large mackintosh sheet is required, big enough to stretch from side to side of the bed, and from the top to well below the bottom. This is laid on the bed under the patient; the head of the bed is raised about a foot upon blocks; attendants hold up the sides and top of the mackintosh to form a sort of trough in which the patient lies; the lower end of the mackintosh passes into a large can or pail or other receiver in which to catch the water. An attendant then pours water from a can over the neck and chest of the patient, whence it runs down over his body and legs, and so into the pail. The temperature of the water may be anything from 90° F. down to 65° F. as desired, or it may be warm to begin with, and each successive canful may be cooler than the last; as many cans of water are thus poured over him as may seem indicated; and then the patient is treated exactly as if he had actually been given a cooled bath. The method is merely a means of giving what amounts to a cool bath when there is no actual bath available for the purpose. It is equally efficient in reducing the pyrexia, and may be repeated as often as required. It has the double disadvantage of needing a very large mackintosh sheet, and of necessitating the presence of several assistants. Nevertheless, there are circumstances in which this method may be gladly adopted by the practitioner.

In the tropics or at sea another form of cold affusion is resorted to, especially in cases of heat-stroke with hyperpyrexia; and this consists in playing cold water all over the patient by means of a spray or hose.

11. *The Continuous Bath*: This is less a treatment for hyperpyrexia when it is present than a prophylactic measure against its developing; it is used mainly in typhoid fever, and even here only by some physicians. A large bath is needed, a constant supply of hot water, and a means of regulating its temperature. The latter should be between 98° F. and 90° F., approaching the lower figure in proportion as the patient's body temperature rises higher. A floating pillow is required to support the patient's back and head without effort, and the patient remains immersed in the bath throughout the high pyrexial part of the illness. Some patients have been kept in the bath continuously in this way for over 30 days; nursing is easy, for all discharges are passed into the bath and removed by the overflow water; the patient sleeps, eats, indeed does everything in the bath until the fever abates. Needless to say, it is only in very special circumstances that this form of treatment is possible, however good the results of it may be. They are said to be very excellent, but the method is only feasible in a hospital or in the homes of the rich.

We have given all the various mechanical means of treating hyperpyrexia, believing that each form may one day or other be of service in the practice of the reader. It is obvious, however, that the simplest forms will be most used in private practice, and these undoubtedly are tepid or cold sponging, ice-bags inside a cradle, and next to these a warm bath in which the patient is immersed whilst the water is allowed to cool.

